# Recombinant CRAMP-producing *Lactococcus lactis* attenuates dextran sulfate sodium-induced colitis by colonic colonization and inhibiting p38/NF-κB signaling

**DOI:** 10.29219/fnr.v65.5570

**Published:** 2021-09-28

**Authors:** Jiahong Li, Shiwen Yu, Xiaohua Pan, Ming Zhang, Zhuwu Lv, Li-Long Pan, Jia Sun

**Affiliations:** 1 State Key Laboratory of Food Science and Technology, Jiangnan University, Wuxi, Jiangsu, P. R. China; 2 School of Food Science and Technology, Jiangnan University, Wuxi, Jiangsu, P. R. China; 3 Department of Obstetrics, Wuxi People’s Hospital Affiliated to Nanjing Medical University, Wuxi, P. R. China; 4 Wuxi School of Medicine, Jiangnan University, Wuxi, Jiangsu, P. R. China

**Keywords:** CRAMP, colitis, Lactococcus lactis NZ9000, Usp45, probiotics

## Abstract

**Background:**

Inflammatory bowel diseases (IBDs) are generally characterized by persistent abdominal pain and diarrhea caused by chronic inflammation in the intestine. Cathelicidins are antimicrobial peptides with pleiotropic roles in anti-infection, wound healing, and immune modulation. However, the sensitivity to the acidic environment and short half-life of cathelicidins limit their application in IBD treatment. Recombinant cathelicidin-related antimicrobial peptide (CRAMP)-producing *Lactococcus lactis* may represent a potential approach for IBD therapy.

**Objective:**

The aim of this study was to develop recombinant CRAMP-producing *L. lactis* NZ9000 and explore the role and mechanism of recombinant *L. lactis* NZ9000 expressing CRAMP in colitis.

**Design:**

We constructed two strains of CRAMP-producing *L. lactis* NZ9000 with different plasmids pMG36e (*L.L*-pMU45CR) or pNZ8148 (*L.L-*pNU45CR), which use a Usp45 secretion signal to drive the secretion of CRAMP. Bacterial suspensions were orally supplemented to mice with a syringe for 4 days after dextran sodium sulfate (DSS) treatment. Body weight change, disease active score, colon length, and colonic histology were determined. The expression of tight junction (ZO-1, ZO-2, and Occludin) and cytokines (IL-6, IL-1β, TNF-α, and IL-10) in colon was performed by qPCR. The expression of p-ERK, p-p38, and p-p65 was determined by Western blot analysis.

**Results:**

Both CRAMP-producing *L. lactis* NZ9000 strains protected against colitis, as shown by reduced weight loss and disease activity score, improved colon shortening, and histopathological injury. In addition, CRAMP-producing *L. lactis* NZ9000 restored gut barrier by upregulating ZO-1, ZO-2, and occludin. Moreover, CRAMP-producing *L. lactis* NZ9000 regulated the colonic cytokines profile with reduced IL-6, IL-1β, and TNF-α production, and increased IL-10 production. By further analysis, we found that CRAMP-producing *L. lactis* NZ9000 reduced the expression of p-p38 and p-p65.

**Conclusions:**

Together, our data suggested that CRAMP-secreting *L. lactis* NZ9000 attenuated dextran sulfate sodium-induced colitis by colonic colonization and inhibiting p38/NF-κB signaling. Orally administered recombinant CRAMP-secreting *L. lactis* NZ9000 represents a potential strategy for colitis therapy.

## Popular scientific summary

- Two strains of recombinant CRAMP-producing *Lactococcus lactis* NZ9000 were constructed with different plasmids pMG36e (*L.L*-pMU45CR) or pNZ8148 (*L.L*-pNU45CR), which use a Usp45 secretion signal to drive the secretion of CRAMP.
- CRAMP-producing *L. lactis* NZ9000 strains protected mice from colitis via suppressed activation of p-p38/NF-κB signaling, thus resulting in a restored cytokines profile and an improved gut barrier integrity.
- CRAMP-producing *L. lactis* NZ9000 represents a novel intervention strategy for colitis treatment.

## Introduction

Inflammatory bowel diseases (IBDs) are inflammatory conditions in the intestine, which generally include ulcerative colitis (UC) and Crohn’s disease (CD) (1). Both UC and CD are known to increase the risk of colorectal cancer (2).The prevailing symptoms of IBDs include abdominal pain, diarrhea, and hematochezia, which have serious negative effects on the quality of life (3). Current therapies of IBD, such as 5-aminosalicylic acid and glucocorticoids, are principally aimed at inhibiting inflammation. However, using anti-inflammatory drugs may result in some side effects, involving diarrhea, headache, and loss of appetite (4, 5). In addition, antibiotics, which are commonly used as adjuvant therapy in IBD, could result in microbiota dysbiosis. Thus, it is necessary to develop a new approach for IBD treatment.

Cathelicidins are antimicrobial peptides found in humans and mice, which includes LL-37 and cathelicidin-related antimicrobial peptide (CRAMP), that are homologous in nature, (6). These have multifaceted roles in wound healing, anti-inflammation, and anti-apoptosis, and have been reported to regulate gut microbes and intestinal homeostasis (7, 8). However, oral administration of LL-37 seems to be impossible due to its sensitivity to acidic environment in gaster (9, 10). CRAMP is also pH sensitive. The α-helical conformation of CRAMP is important for its bioactivity, and this can be lost at low pH conditions. The protonation of acidic side chains results in the loss of stabilizing complementary side chain ion pairs (10–12). However, its short half-life (1 h in cells) also limits systemic administration (13). Therefore, new cathelicidins-targeted approaches are necessary in the treatment of colitis.

The use of probiotics is a potential approach to treat or prevent IBD. Although most clinical trials indicate that probiotics are beneficial in treating IBD, in some cases, probiotics induce intestinal damage or bacteremia (14, 15). *Lactococcus lactis* (*L. Lactis*) is widely used in food fermentation and oral delivery of therapeutic proteins (16). *Lactococcus lactis* NZ9000 (*L. Lactis* NZ9000) plays a key role in industrial fermentation (17) and has been used to develop genetically modified microorganisms for the treatment of colitis in mice (18, 19).

In this study, we constructed two recombinant *L. lactis* NZ9000 strains, which used a Usp45 secretion signal to drive the secretion of CRAMP, with pMG36e (*L.L*-pMU45CR) or pNZ8148 (*L.L* pNU45CR) plasmids, respectively. We also evaluated the role and mechanism of CRAMP-secreting *L. lactis* NZ9000 in experimental colitis.

## Materials and methods

### Plasmids, bacterial strains, and growth conditions

*Lactococcus lactis* NZ9000 (NIZO Food Research, Kernhemseweg, the Netherlands) was grown in M17 medium (Solarbio, Beijing, China) containing 0.5% (w/v) glucose (Solarbio, Beijing, China) at 30°C. CRAMP gene (20) containing a Usp45 secretion signal gene (21) was synthesized by Sangon Biotech Co., Ltd (Shanghai, China) and was cloned into pMG36e (22) (pMG36e-Usp45-CRAMP) and pNZ8148 (23) (pNZ8148-Usp45-CRAMP) at the Xbal/Sphl sites, respectively.*Lactococcus lactis* NZ9000 was transformed with pMG36e-Usp45-CRAMP (*L.L-*pMU45CR) and pNZ8148-Usp45-CRAMP (*L.L-​*pNU45CR) by electroporation (24). *Lactococcus lactis* NZ9000 was transformed with empty pMG36e (*L.L-*pMVectro) and empty pNZ8148 (*L.L-*pNVector) as controls. Nisin (1.25 ng/mL, Solarbio, Beijing, China) was used for 4 h to induce gene expression in *L.L-*pNU45CR and *L.L-*pNVector as controls (19). Bacterial suspensions were prepared freshly and were orally administered with syringe once daily for 4 days after dextran sodium sulfate (DSS) treatment. DSS was purchased from MP Biomedicals (Irvine, CA, USA), with a molecular weight of 36–50 kDa.

### Mice

Male C57BL/6 mice (7–8 weeks old) were purchased from Su Pu Si Biotechnology Co. Ltd (Suzhou, Jiangsu, China) and were randomly divided into six groups (*n* = 5) as follows: [1] control (without DSS), [2] 3% DSS + sterilized water, [3] 3% DSS + *L.L-*pMVector, [4] 3% DSS + *L.L*-pNVector, [5] 3% DSS + *L.L-*pMU45CR, and [6] 3% DSS + *L.L-* pNU45CR. After 7 days of DSS treatment, bacterial suspensions (10^10^ CFU) were orally administrated with syringe once daily for 4 days. Bacterial suspensions were prepared as described below. The bacterial cells were centrifuged at 3,000 g at 4°C for 20 min and washed twice with PBS. These bacterial cells were resuspended at concentrations of 5 × 10^10^ CFU/mL based on optical density (OD^600^). Mice were gavaged with 200 µL of bacterial suspension (10^10^ CFU) with syringe. To check the number of CFUs, plate counts were performed on M17 agar supplemented with 0.5% glucose at 30°C without agitation. After 24 h, the plates were observed.

### Disease activity index (DAI) score

The degrees of bleeding, body weight change, and stool consistency were recorded to determine the disease activity score as previously described (25, 26): bleeding (score 0, normal; score 1, stool hemoccult positive; score 2, hemoccult positive and visual pellet bleeding; and score 4, gross bleeding, blood around anus), body weight loss (score 0, no weight loss; score 1, weight loss within 1–5%; score 2, within 5–10%; and score 3, >10%), and stool consistency (score 0, normal; score 1, soft but firm; score 2, soft; and score 3, diarrhea).

### ELISA

Colon tissues were homogenized, and the expression of CRAMP was analyzed with an ELISA kit (CUSABIO TECHNOLOGY LLC, Wuhan, Hubei, China) according to the manufacturer’s instructions.

### Histology and immunostaining

Colon tissues were fixed and embedded in paraffin via standard methods (27), and sectioned into 5 µm slices and stained with hematoxylin-eosin (Yulu, Nanchang, Jiangxi, China). Histological scoring was performed as follows: epithelium (score 0, normal; score 1, crypt loss <10%; score 2, crypt loss 10–50%; score 3, crypt loss 50–90%; score 4, crypt loss >90%; score 5, ulcer 1–50%; and score 6, ulcer >50%). Infiltration for mucosa (score 0, normal; score 1, <10%; score 2, 10–50%; and score 3, >50%), submucosa (score 0, normal; score 1, 1–50% and score 2, >50%), and muscle or serosa (score 0, normal and score 1, >1%). Histological scores were sum of epithelial damage scores and inflammatory cell infiltration scores (28, 29). Immunostaining was performed as described in our previous study (30, 31). For immunofluorescent staining, anti-ZO-1 (1:100, A11417, ABclonal Technology Co., Ltd, Wuhan, Hubei, China) and anti-rabbit Alexa Fluor 555 (1:500, A32732, Invitrogen, Carlsbad, CA, USA) were used. For nucleic acid staining, DAPI (Beyotime Biotechnology, Shanghai, China) was used according to the manufacturer’s instructions.

### Real-time PCR

TRIzol reagent (CoWin Bioscience Co., Ltd, Beijing, China) was used for colonic RNA isolation. SuperRT cDNA synthesis kit (CoWin Bioscience Co., Ltd, Beijing, China) was used for reverse transcription. SYBR Green (CoWin Bioscience Co., Ltd, Beijing, China) was used for quantitation. The 2^-∆∆ct^ method was used for calculation and normalized to β-actin. All primers were synthesized by Thermo Fisher Scientific (Waltham, MA, USA). Primers were designed with Primer 5 software (Premier Biosoft, Palo Alto, CA, USA). To check the specificity of primers, blast program (32) was used, and PCR products were detected by agarose gel electrophoresis. Primers used for qPCR are shown in Table 1.

**Table 1:** Primers used for qPCR.

Gene	Forward (5’-3’)	Reverse (5’-3’)
ZO-1	GCCGCTAAGAGCACAGCAA	TCCCCACTCTGAAAATGAGGA
ZO-2	ATGGGAGCAGTACACCGTGA	TGACCACCCTGTCATTTTCTTG
Occludin	TTGAAAGTCCACCTCCTTACAGA	CCGGATAAAAAGAGTACGCTGG
IL-6	GAGTCACAGAAGGAGTGGCTAAGG	CGCACTAGGTTTGCCGAGTAGATCT
IL-1β	TTCAGGCAGGCAGTATCACTC	GAAGGTCCACGGGAAAGACAC
TNF-α	CCACGCTCTTCTGTCTACTG	ACTTGGTGGTTTGCTACGAC
IL-10	GGACCAGCTGGACAACATACTGCTA	CCGATAAGGCTTGGCAACCCAAGT
β-Actin	GGCTGTATTCCCCTCCATCG	CCAGTTGGTAACAATGCCATGT

### Western bolt

To compare the expression of secreted or intracellular CRAMP, Supernatant and cell fractions were prepared as described by Le Loir et al (33). Briefly, 2 mL of *L. Lactis* cultures at a given optical density of 600 nm (OD^600^) were harvested by centrifugation at 3,000´g for 20 min at 4°C. The equivalent of 1 mL of 1 OD^600^ unit of culture (cell or supernatant) was concentrated in a 100 μL final volume as described below, and 10 μL was loaded for SDS-PAGE. Supernatants were precipitated by the addition of 10% trichloroacetic acid, harvesting by centrifugation at 10,000 × g at 4°C. The resulting pellet was dissolved in a 1:20 volume of 50 mM NaOH. Cell pellets were resuspended in 70 μL of TES containing lysozyme (1 mg/mL). After 30 min of incubation at 37°C, cells were lysed with 30 μL of 20% SDS. Equal volumes of 2× loading buffer were added to all samples. RIPA buffer with phosphatase and protease inhibitors was purchased from Songon Biotech (Shanghai, China) and used in the preparation of colon samples. Electrophoresis and transfer were performed as described before (34). Primary antibodies were obtained from CST (Danvers, MA, USA): p-p38 (#4511), p-Erk1/2 (#9101), p-NF-κBp65 (#3033), total-p38 (#8690), total-ERK (#4695), and total-NF-κB p65 (#4674) were used. Anti-rabbit secondary antibody was purchased from Thermo Fisher Scientific (31460, Waltham, MA, USA). An enhanced chemiluminescent (ECL) kit (Millipore, Billerica, MA, USA) was used for detection.

### Statistical analysis

The data were expressed as mean ± standard error of mean (SEM) and statistically analyzed by GraphPad Prism software (San Diego, CA, USA) using one-way analysis of variance followed by Tukey’s post-hoc test. *P* ≤ 0.05 was considered to be statistically significant.

## Results

### Construction of recombinant CRAMP-secreting L. lactis NZ9000

**Fig. 1:**
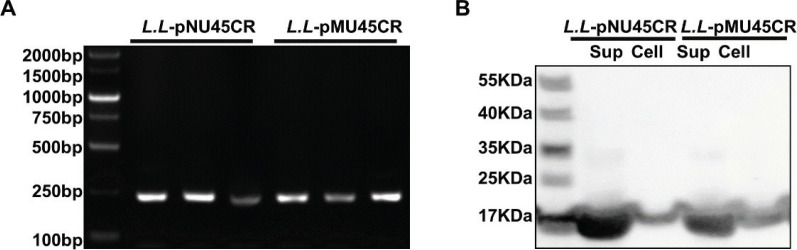
Construction of CRAMP-secreting *L. lactis* NZ9000. (A) Polymerase chain reaction (PCR) products of CRAMP connected with a Usp45 signal peptide (Usp45 + CRAMP) in *L.L*-pMU45CR and *L. L*-pNU45CR were determined by agarose gel electrophoresis. *L. L*-pMU45CR was based on pMG36e plasmids, and *L. L*-pNU45CR was based on pNZ8148 plasmids. The product size of Usp45 + CRAMP is 243 bp. (B) Protein expression of CRAMP in recombinant bacterial cells and supernatant (Sup) was examined by Western blotting.

Single colonies were picked and detected by polymerase chain reaction (PCR) to confirm whether CRAMP with secretion signal peptides (Usp45 + CRAMP) were expressed in *L.L*-pMU45CR or *L.L-*pNU45CR. As shown in Fig. 1A, Usp45 + CRAMP was expressed in recombinant bacterial cells. In addition, Western blot analysis showed that both *L.L*-pMU45CR and *L.L*-pNU45CR expressed CRAMP, and the levels of CRAMP were higher in supernatants than in bacterial cells (Fig. 1B). Collectively, these date data revealed that CRAMP was expressed in recombinant *L.L*-pMU45CR and *L.L*-pNU45CR strains, and both strains have higher levels of secreted CRAMP compared with intracellular CRAMP.

### Recombinant CRAMP-producing L. lactis NZ9000 alleviates dextran sodium sulfate-induced colitis

**Fig. 2:**
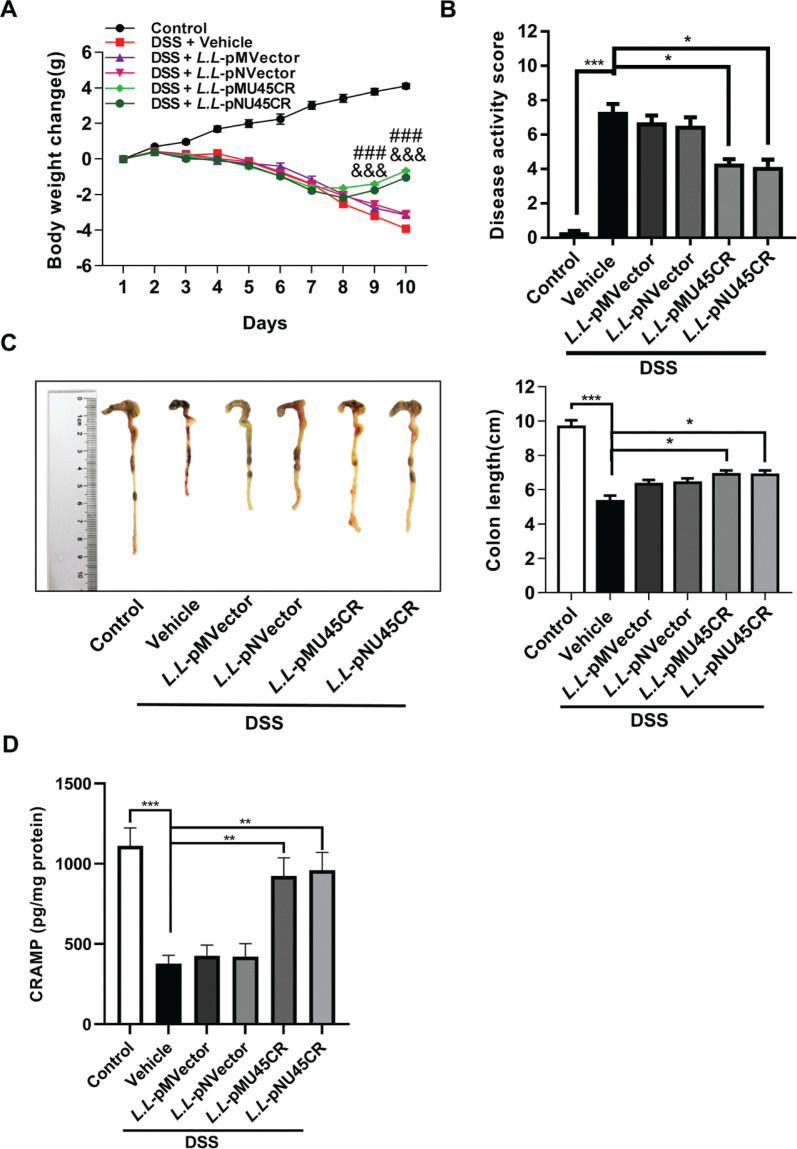
Recombinant CRAMP-producing *L. lactis* NZ9000 attenuates dextran sodium sulfate-induced colitis. (A) Weight change was recorded every day (g); (B) comparison of disease activity score on day 10; (C) colon length was measured on day 10. *L.L*-pMVector is recombinant *L. lactis* NZ9000 with pMG36e empty vector and *L. L*-pNVector is recombinant *L. lactis* NZ9000 with pNZ8148 empty vector. (D) CRAMP expression in colonic tissues was detected by ELISA. Data are expressed as means ± SEM, *n* = 5. ^###^*P* < 0.001 compared between Vehicle and *L. L*-pMU45CR group in Fig. 2A; ^&&&^*P* < 0.001 compared between Vehicle and *L. L*-pNU45CR group in Fig. 2A; *P < 0.05, ***P < 0.001.

To address the protective effect of recombinant CRAMP-producing *L. lactis* NZ9000 on colitis, we treated mice with recombinant *L. lactis* NZ9000 for 4 days after dextran sodium sulfate (DSS)-induced colitis. We found that both *L.L*-pMU45CR and *L.L*-pNU45CR attenuated colitis in DSS-treated mice, as shown by reduced weight change (Fig. 2A) and disease activity score (Fig. 2B), which consists of stool consistency, body weight change and bleeding, and improved colon length (Fig. 2C). Furthermore, we found that two recombinant CRAMP producing *L. lactis* strains restored the expression of CRAMP, which was suppressed by DSS-induced colitis (Fig. 2D).

### Recombinant CRAMP-producing L. lactis NZ9000 improves crypt morphology in colitis mice

**Fig. 3:**
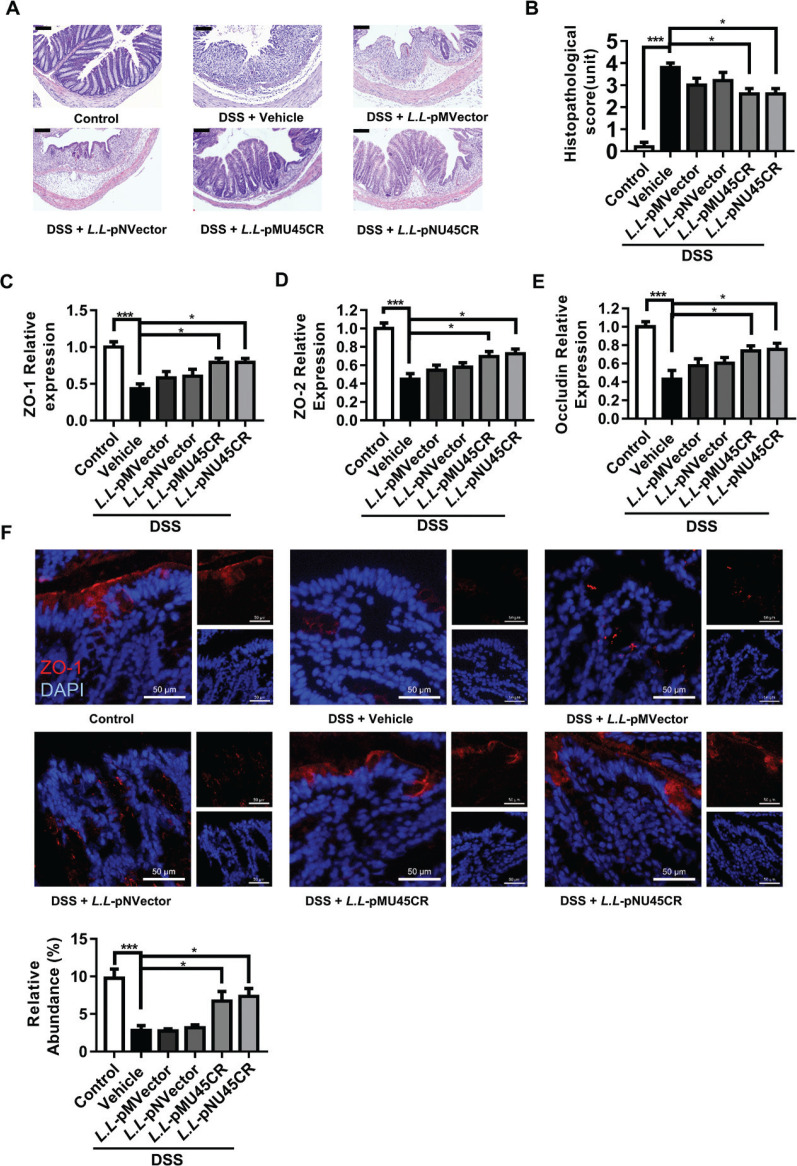
Recombinant CRAMP-producing *L. lactis* NZ9000 improves crypt morphology in colitis. (A) Histological analysis was performed by hematoxylin-eosin (H&E) staining in colonic tissues. The scale bar is 100 μm; (B) the histological score of colon; relative mRNA levels of tight junctions in colon were determined by qPCR (C) ZO-1, (D) ZO-2, and (E) occludin. ZO-1, tight junction protein 1; ZO-2, tight junction protein 2. (F) Representative graphs showing immunofluorescence of ZO-1 (red) in colonic tissues (DAPI, blue). Scale bar: 50 μm. Data are expressed as means ± SEM, *n* = 5. *P < 0.05, ***P < 0.001.

By further histological examination, we found that CRAMP-encoding *L. lactis* NZ9000 resulted in reduced colonic mucosal injury, crypt destruction, and inflammatory cell infiltration than in vehicle-treated mice (Fig. 3A–B). In addition, CRAMP-secreting *L. lactis* NZ9000 restored the gut barrier, as shown by increased ZO-1 (Fig. 3C), ZO-2 (Fig. 3D), and occludin (Fig. 3E) expression. Consistently, we found ZO-1 were expressed in apical border under steady state. And two recombinant CRAMP producing *L. lactis* strains prevented the loss of apical ZO-1 in epithelium (Fig. 3F). Together, these data indicated that recombinant CRAMP-producing *L. lactis* NZ9000 reduced histological damage and tight-junction disruption.

### Recombinant CRAMP-producing L. lactis NZ9000 promotes a modulatory cytokine profile in colitis

**Fig. 4:**
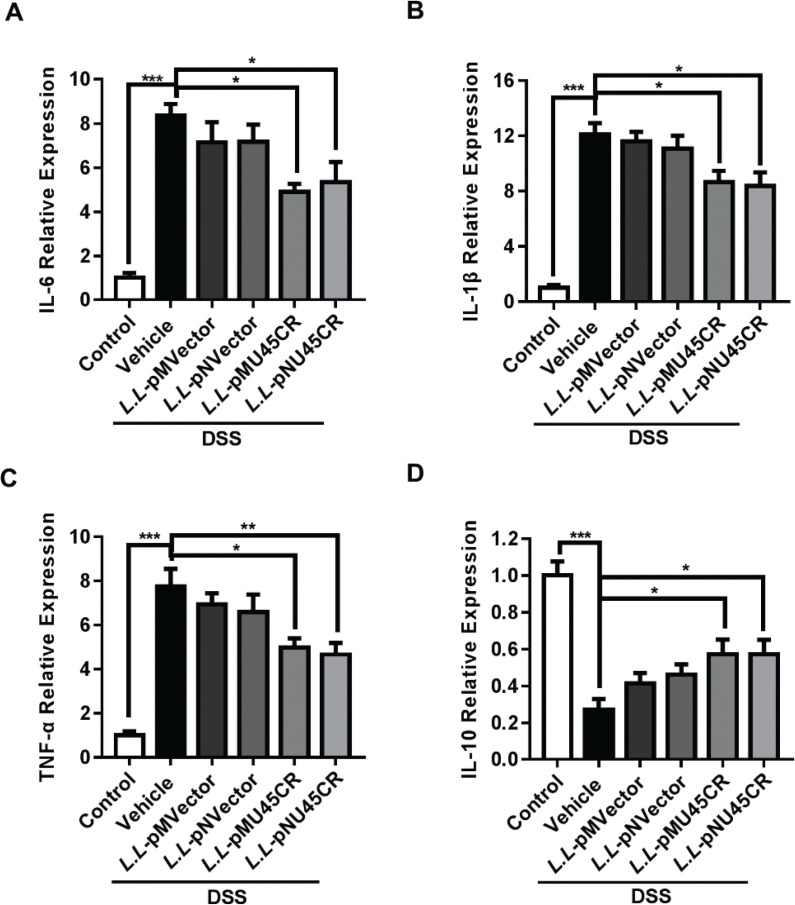
Recombinant CRAMP-producing *L. lactis* NZ9000 promotes a modulatory cytokine profile in colitis. Relative expression of (A) IL-6, (B) IL-1β, (C) TNF-α and (D) IL-10 in colon was determined by qPCR. IL-6, interleukin-6; IL-1β, interleukin-1beta; TNF-α, tumor necrosis factor alpha. Data are expressed as means ± SEM, *n* = 5. *P < 0.05, **P < 0.01, ***P < 0.001.

During colitis development, the production of cytokines was a central event. We detected several key cytokines in colitis. We found that treatment of both *L.L*-pMU45CR and *L.L*-pNU45CR reduced IL-6 (Fig. 4A), IL-1β (Fig. 4B), and TNF-α (Fig. 4C) expression, and restored IL-10 production (Fig. 4D) which plays a regulatory role in colitis. Collectively, these data revealed that both *L.L*-pMU45CR and *L.L*-pNU45CR treatment promotes a modulatory cytokine profile in DSS-induced colitis.

### Recombinant CRAMP-producing L. lactis reduces the expression of p-p38 and p-p65

**Fig. 5:**
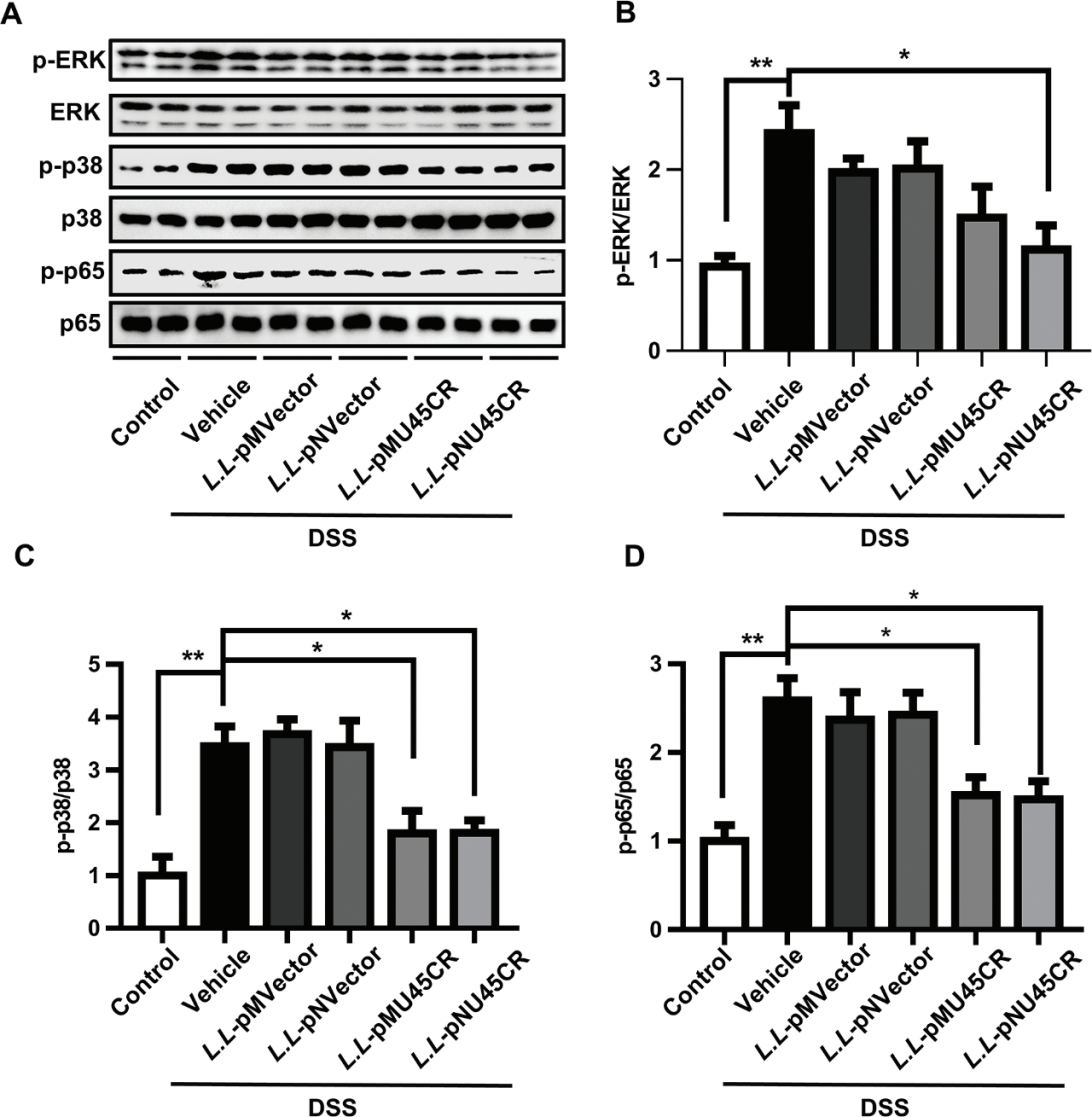
Recombinant CRAMP-producing *L. lactis* reduces the expression of p-p38 and p-p65. (A) Expressions of p-ERK, p-p38 and p-p65 in colons were determined by Western blot analysis. Total ERK, p38, and p65 were used as loading controls, and the gray value of each band (B–D) was normalized to loading controls. Data are expressed as means ± SEM, *n* = 5. *P < 0.05, **P < 0.01.

To further explore the underlying mechanism of *L.L*-pMU45CR and *L.L*-pNU45CR treatment in DSS-induced colitis, we next detected several key transcription factors in colons. As shown in Fig. 5A, we found that *L.L-pMU45CR* treatment significantly reduced the expression of p-p38 and p-p65, and *L.L-pNU45CR* treatment reduced the expression of p-ERK, p-p38, and p-p65 (Fig. 5A). Collectively, these data indicated that recombinant CRAMP-producing *L. lactis* inhibited colon inflammation by suppressing p38 and p65 activation.

## Discussion

In this study, we developed two strains of CRAMP-producing *L. lactis* NZ9000, which use a Usp45 secretion signal to drive the secretion of CRAMP. The results of this study indicate that orally administered recombinant CRAMP-producing *L. lactis* protected mice from colitis by suppressing the expression of p-p38 and p-p65 and subsequently decreasing the expression of proinflammatory cytokines, thus improving the integrity of gut barrier.

*Lactococcus lactis* is suitable for expressing bioactive molecules in IBD therapy. Most (90–98%) *L. lactis,* which transit with diet, could survival through acidic conditions in gastric juice (16). Recombinant *L. lactis* NZ9000 is able to colonize the colon and has been used to express bioactive molecules in the treatment of colitis, such as theme oxygenase-1 and insulin-like growth factor I (18, 19). The secretion efficiencies of heterologous proteins in recombinant *L. lactis* are varied due to the characteristics of heterologous proteins. Usp45 is a signal peptide widely applied in driving protein secretion (35). We achieved higher levels of secreted CRAMP in recombinant *L. lactis* NZ9000 with Usp45 signal peptides.

Cathelicidins have multifunctional roles, such as anti-microbial, anti-inflammation, anti-apoptosis activities, and wound healing. Serum LL-37 levels are positively correlated with recovery in IBD patients (36). We observed elevated CRAMP expression in the colon of recombinant CRAMP producing *L. Lactis*-treated mice. However, the recombinant CRAMP producing *L. lactis* may not have a direct effect on serum CRAMP, because bioactive peptides were not able to cross the gut wall intact, except dipeptides and tripeptides (37). CRAMP has been reported to protect mice from colitis by resisting bacteria invasion into colonic tissues and preventing colonic inflammation (38, 39). Consistent with our results, CRAMP has been reported to suppress the expression of p-p38 and p-p65, thus alleviating inflammation (40, 41).

Cytokines exert major impacts on intestinal inflammation and related clinical symptoms in IBD. The unbalanced cytokines profile between proinflammatory and regulatory cytokines promotes mucosal inflammation. IL-10 regulates intestinal homeostasis, and its deficiency leads to spontaneous colitis in mice (42). We found that recombinant CRAMP-producing *L. lactis* increased the expression of IL-10. Similarly, cathelicidins increased the expression of IL-10 in human mononuclear cells (43, 44). Blockade of proinflammatory cytokines, such as TNF-α, is recognized as a crucial strategy for IBD therapy (1). Cathelicidins were shown to reduce TNF-α expression in macrophages by suppressing the expression of p-p38 and p-p65 (40, 45, 46). Similarly, we found that both *L.L*-pMU45CR and *L.L*-pNU45CR reduced IL-6, TNF-α, and IL-1β expression by suppressing the expression of p-p38 and p-p65. These results revealed that recombinant CRAMP-producing *L. lactis* regulates proinflammatory cytokine expression by p-p38/NF-κB p-p65 signaling.

Epithelial tight junctions are crucial in regulating intestinal barrier and permeability. Disruption of intestinal barrier results in the transfer of intestinal bacteria and antigens into submucosa, thus subsequently leading to inflammatory response, such as transcription factor activation and immune cell infiltration. Tight junctions are regulated by intestinal microbes and cytokines in IBD. Adherent-invasive *Escherichia coli* is increased in IBD patients and can disrupt tight junctions (47). Several cytokines, such as TNF-α, IL-1β, and IL-6, disrupt tight junctions and IL-10 restores tight junctions (48). Previous studies have reported that cathelicidins upregulated tight junctions in bronchial epithelium (49) and epidermal keratinocyte (50). Together, the results of this study indicated that recombinant CRAMP-producing *L. lactis* restored the expression of tight junctions by regulating the cytokines profile in colitis, although it is unclear whether recombinant CRAMP-producing *L. lactis* regulates adherent-invasive *E. coli*.

## Conclusions

**Fig. 6:**
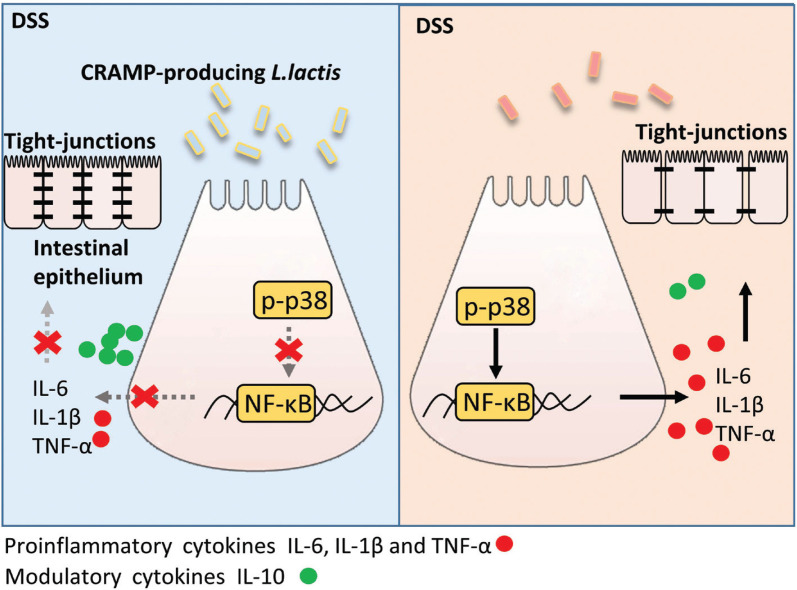
Graphical summary for the role and mechanism of recombinant CRAMP-producing *L. lactis* in colitis. CRAMP-secreting *L. lactis* NZ9000 strains protected mice from colitis via suppressed activation of p-p38/NF-κB p-p65 signaling, thus resulting in a restored cytokines profile and an improved gut barrier integrity.

Our data revealed that two CRAMP-secreting *L. lactis* NZ9000 strains protected mice from colitis via suppressed activation of p-p38/NF-κB p-p65 signaling, thus resulting in a restored cytokines profile and improved gut barrier integrity (Fig. 6). Our data suggested that orally administered CRAMP-secreting *L. lactis* NZ9000 may represent a potential strategy for IBD therapy.
